# Anesthetic management of one-stage scheduled surgery for adrenal cortical carcinoma complicated by massive pulmonary tumor embolism

**DOI:** 10.1186/s40981-017-0115-4

**Published:** 2017-09-02

**Authors:** Kazuma Yunoki, Tsutomu Wada, Ikuko Miyawaki, Kazuo Yamazaki, Hiroyuki Mima

**Affiliations:** 1 The Department of Anesthesia and Critical Care, Kobe City Medical Center General Hospital, 2-1-1, Minatojimaminamimachi, Chuo-ku, Kobe city, Hyogo 6500047 Japan; 2 The Department of Anesthesiology, Rinku General Medical Center, Rinku Ourai Kita 2-23, Izumisano city, Osaka, 5988577 Japan

**Keywords:** Adrenal cortical carcinoma, Cardiopulmonary bypass, Inferior vena cava, One-stage surgery, Pulmonary tumor embolism, Transesophageal echocardiography

## Background

Adrenal cortical carcinoma (ACC) with pulmonary tumor embolism (PTE) occurs extremely infrequently, and no case reports are available concerning one-stage scheduled resection of ACC and PTE with cardiopulmonary bypass (CPB). Herein, we report a successful management of patient with ACC complicated by a massive PTE.

## Case presentation

**Fig. 1:**
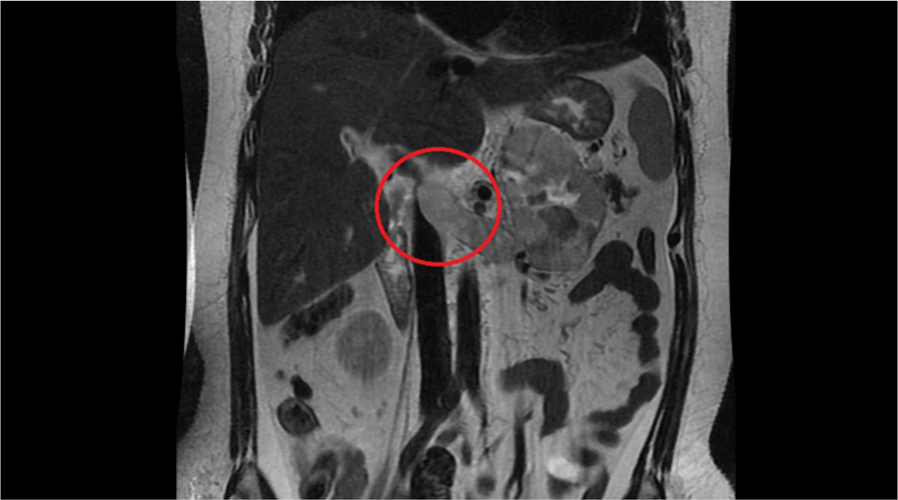
Abdominal magnetic resonance imaging. T1-weighted magnetic resonance image revealing a large (10.0 cm) tumor in the left adrenal gland extending into the inferior vena cava (circle) with the proximal position at the level of the hepatic vein

**Fig. 2:**
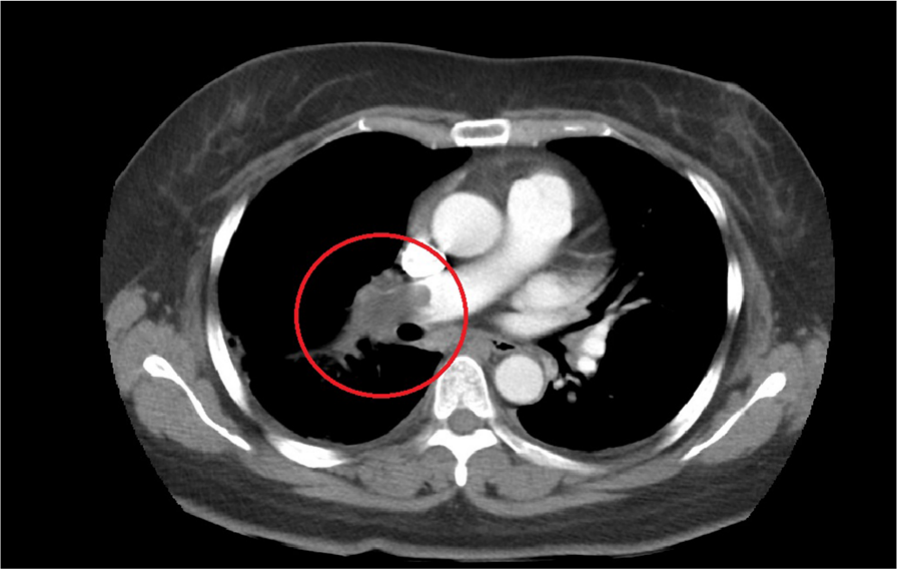
Thoracic contrast-enhanced computed tomography. Contrast-enhanced computed tomography scan revealing a massive pulmonary tumor embolism (circle) occluding the right pulmonary artery

A 44-year-old, 150.0 cm, 85.0 kg female was transferred to our hospital complaining of dyspnea. T1-weighted magnetic resonance imaging revealed a large left adrenal tumor extending into the IVC with its proximal position at the level of the hepatic vein (Fig. 1), and contrast-enhanced CT revealed a massive PTE occluding the right pulmonary artery (PA) (Fig. 2). Transthoracic echocardiography (TTE) revealed moderate tricuspid regurgitation (TR) and a diastolic D-shaped left ventricle. IVC filter placement was not performed due to technical difficulties. Laboratory test results revealed elevated levels of serum cortisol and reduced levels of serum adrenocorticotropic hormone. These findings were suggestive of an ACC. Surgical treatment was considered necessary for the prevention of a lethal PTE, and one-stage surgery was scheduled. This procedure included a left nephrectomy, resection of the IVC tumor thrombus, and removal of the PTE under CPB.

**Fig. 3:**
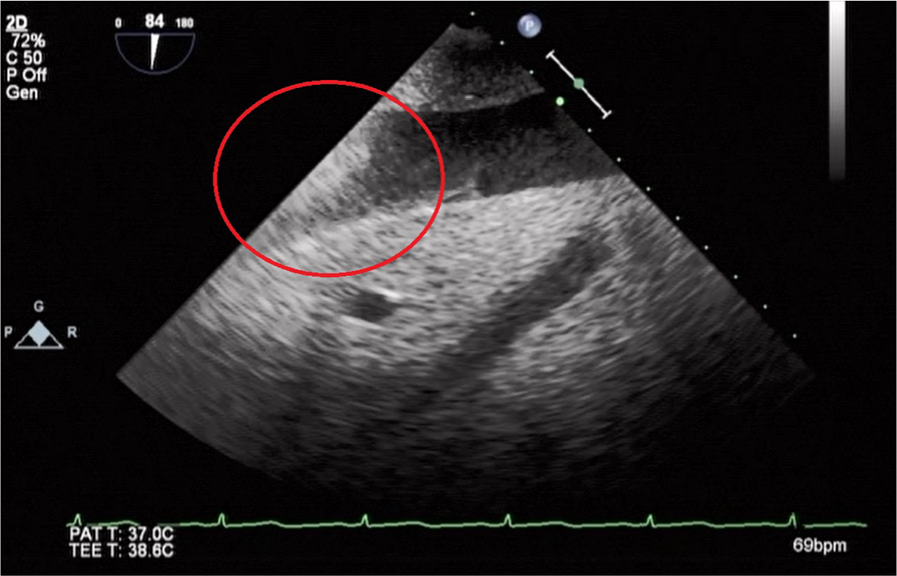
Intraoperative transthoracic echocardiography. Intraoperative transthoracic echocardiography image of the modified transgastric hepatic vein view revealing that the inferior vena cava was almost fully occluded (circle) by a tumor embolus at the level of the inferior hepatic vein

General anesthesia was induced with Propofol, rocuronium, and fentanyl and was followed by endotracheal intubation. After the placement of a transesophageal echocardiography (TEE) probe (Fig. 3) and left radial arterial catheter, a pulmonary artery catheter (PAC) were inserted from the right internal jugular vein and placed with its tip temporarily in the right atrium. A median sternotomy was initially performed for the preparation of an emergent CPB and was followed by abdominal incision. After IVC clamping at the proximal level of the abdominal IVC, the tumor mass was removed coupled with the left adrenal tumor, left renal vein, and IVC tumor thrombus. The hemostasis procedure was a bit challenging due to the bleeding from the swollen abdominal vessels surrounding the tumor. In total, 2800 mL of blood was lost during the abdominal procedure.

After the confirmation that abdominal hemostasis had been complete, PTE resection started. After systemic heparinization, followed by cannulation of the ascending aorta, a total flow bypass with superior vena cava (SVC) and IVC cannulation was established. Significant abdominal bleeding was not observed. PA tumor embolectomy was completed under cardiac arrest, and the tip of the PAC was manually guided into the main PA. The patient was successfully weaned from the CPB. The total aortic cross-clamping and CPB times were 38 and 97 min, respectively. Pulmonary arterial pressure (PAP) was maintained within normal limits. The total duration of the operation was 13 h and 55 min. The total amount of bleeding was 4900 mL, and 1960 mL of red blood cell products were infused. The patient was extubated on the next day and was discharged from hospital on postoperative day 16, without any complications.

## Discussion

One in 10 patients with renal cell carcinoma (RCC) are reported to present with IVC tumor invasion. Of these, 1.0% present with right atrial invasion [1, 2]. In comparison, ACC is a relatively rare cancer and ACC with IVC extension occurs extremely infrequently. Surgical resection is the first choice of treatment if complete removal of the tumor is expected [3]. In our case, though radical surgical removal of the tumor was considered infeasible, surgical treatment was selected to prevent lethal PTEs.

The initial concern regarding this procedure is hemorrhaging. IVC obstructive tumors tend to develop collateral veins around the tumor, and surgical hemostasis is often challenging. Complete hemostasis is, however, required in the abdominal procedure, because the patient is to be heparinized for CPB. If abdominal hemostasis is incomplete, we must consider postponing the pulmonary embolectomy. Furthermore, performing CPB in as short a time as possible and preventing hypothermia may contribute to the preservation of platelet function and the coagulation system.

The second concern is the unanticipated formation of new PTEs. While no reports have been published concerning the risk of PTEs in ACC patients, this case suggests ACC with IVC invasion can cause massive PTESs. So, we should be prepared for abrupt migration of tumor in the case of ACC. The importance of TEE for IVC invading tumors is well recognized [4], and TEE monitoring is recommended for stages 3 and 4 patients [5]. Our patient was classified as stage 2 disease, and TEE monitoring is usually not recommended. Given that the risk of intraoperative tumor migration is still unknown in the case of ACC, however, TEE observation should be necessary even though this is classified as stage 2 in the case of ACC.

## Conclusion

One-stage scheduled surgery for resection of ACC and PTE under CPB was performed successfully. Intraoperative TEE is a useful monitor since the risk of acute PTE by migration of the tumor has yet to be determined in the case of ACC.

## Abbreviations

ACC: Adrenal cortical carcinoma
CPB: Cardiopulmonary bypass
CT: Computed tomography
ICU: Intensive care unit
IVC: Inferior vena cava
PA: Pulmonary artery
PAC: Pulmonary artery catheter
PAP: Pulmonary arterial pressure
PTE: Pulmonary thromboembolism
PV: Pulmonary vein
RCC: Renal cell carcinoma
SVC: Superior vena cava
TEE: Transesophageal echocardiography
TR: Tricuspid regurgitation
TTE: Transthoracic echocardiography
